# Supplementary material for: Relaxed targeting rules help PIWI proteins silence transposons
Source: Nature. 2023 Jun 21;619(7969):394–402. doi: 10.1038/s41586-023-06257-4 (PMC10338343; doi:10.1038/s41586-023-06257-4)
Supplement: Supplementary file 1 — Supplementary Figs. 1–5. Uncropped gel source data. Binding affinities and cleavage rates for MILI, MIWI and EfPiwi. FACS gating strategy to purify mouse primary germ cells. [file 41586_2023_6257_MOESM1_ESM.pdf]

nature portfolio

https://doi.org/10.1038/s41586-023-06257-4

# Supplementary information

# Relaxed targeting rules help PIWI proteins silence transposons

In the format provided by the authors and unedited

Extended Data Fig. 1c

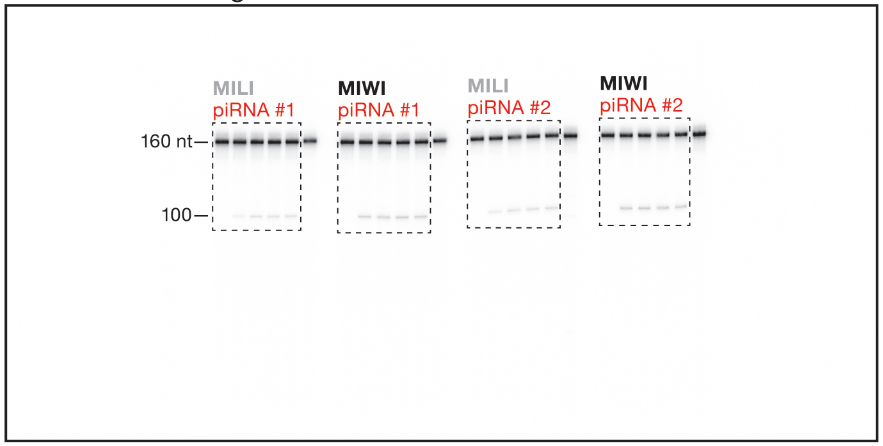


Extended Data Fig. 1c

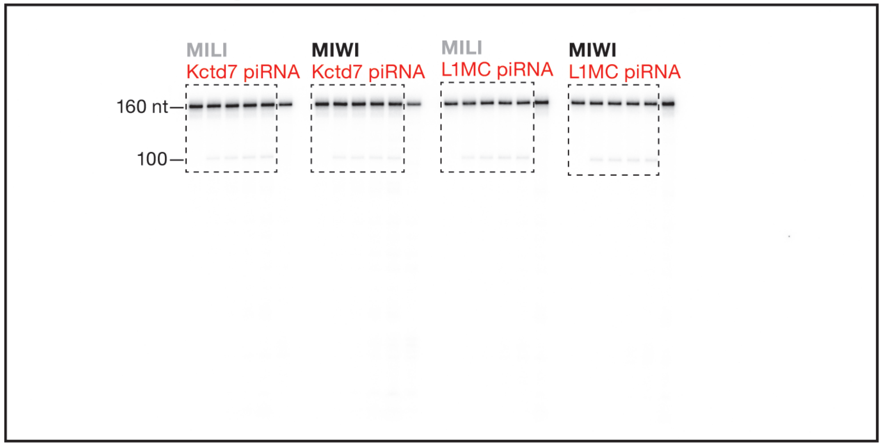


Extended Data Fig. 1c

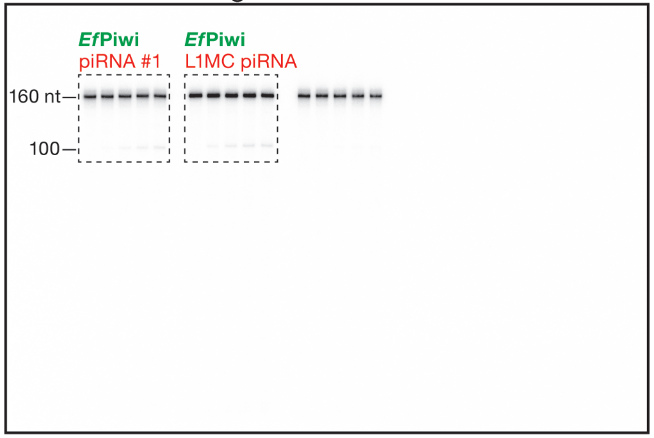


Extended Data Fig. 1d

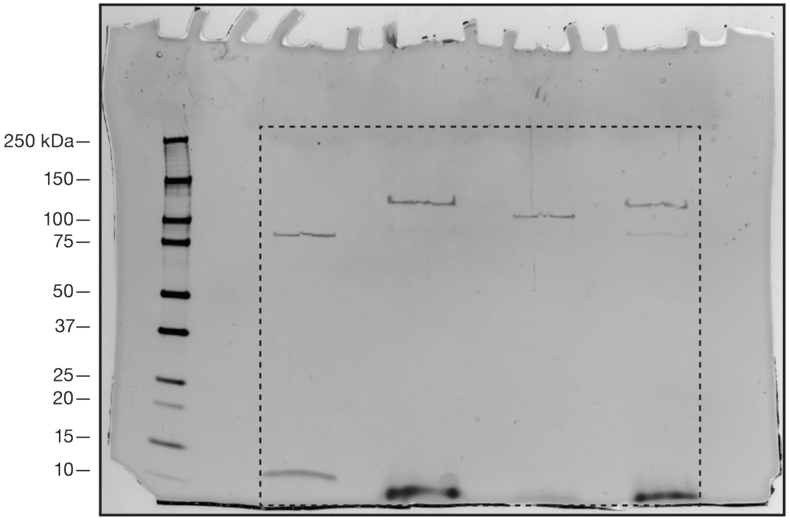


Extended Data Fig. 1e

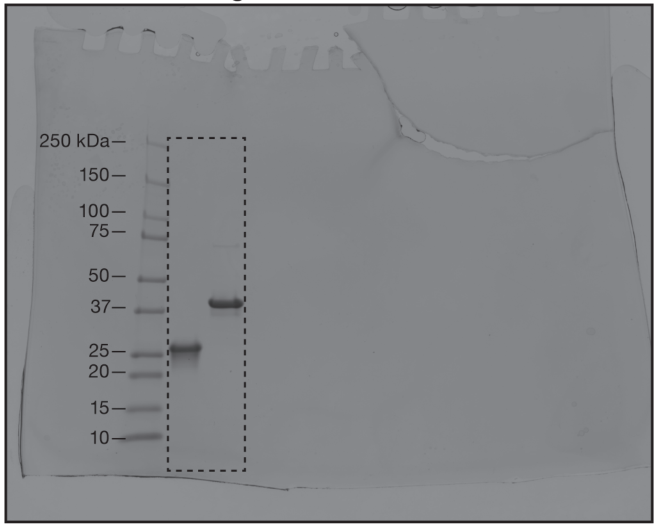


**Supplementary Figure 1.** Uncropped gel source data.

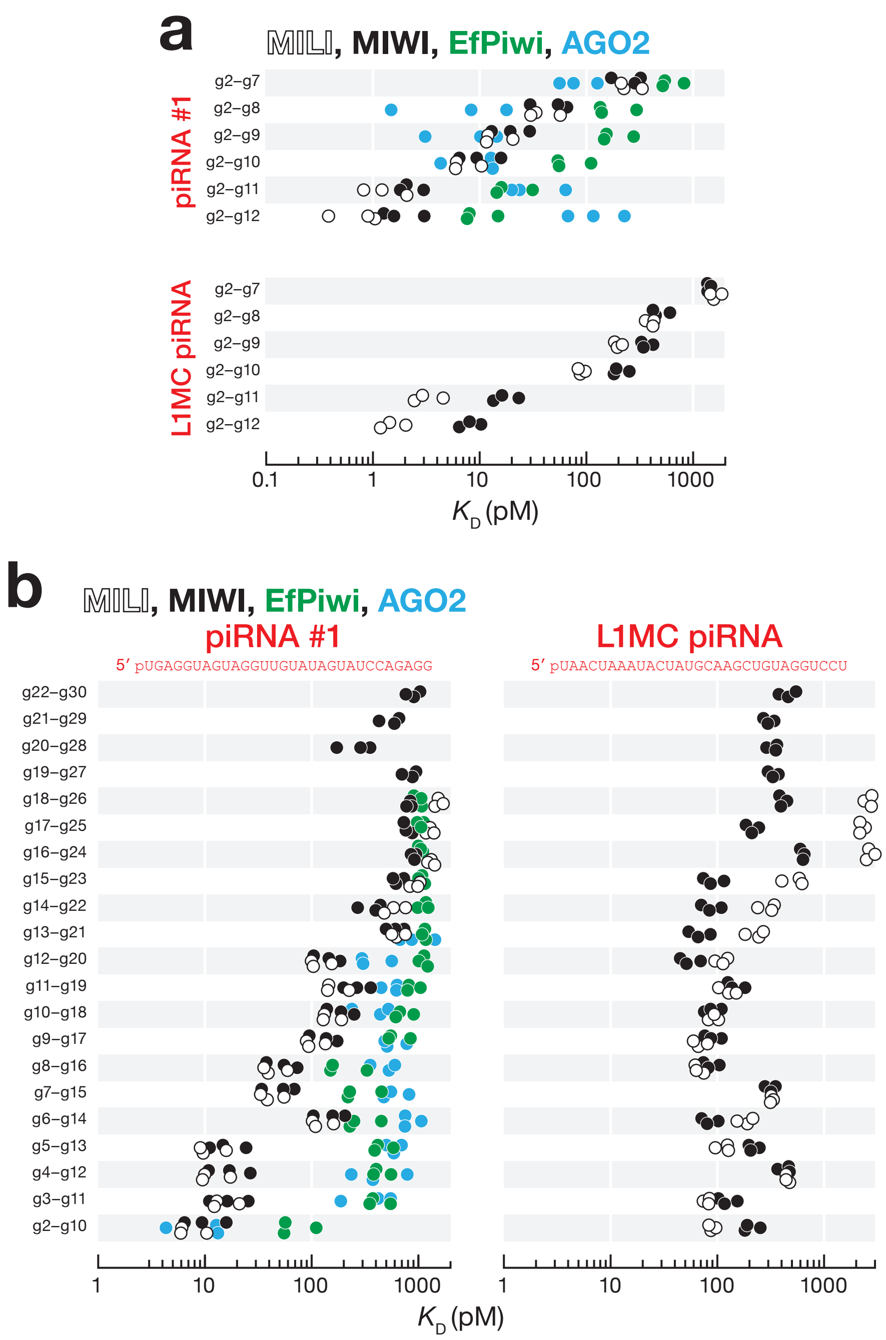


**Supplementary Figure 2.** Binding affinities ($K_D$) of MIWI, MILI, EfPiwi, and mouse AGO2 for canonical (a) and non-canonical (b) target sites. Data are from three independent trials. Mean and the standard deviation of the data are shown in Figs. 1c and 1d.

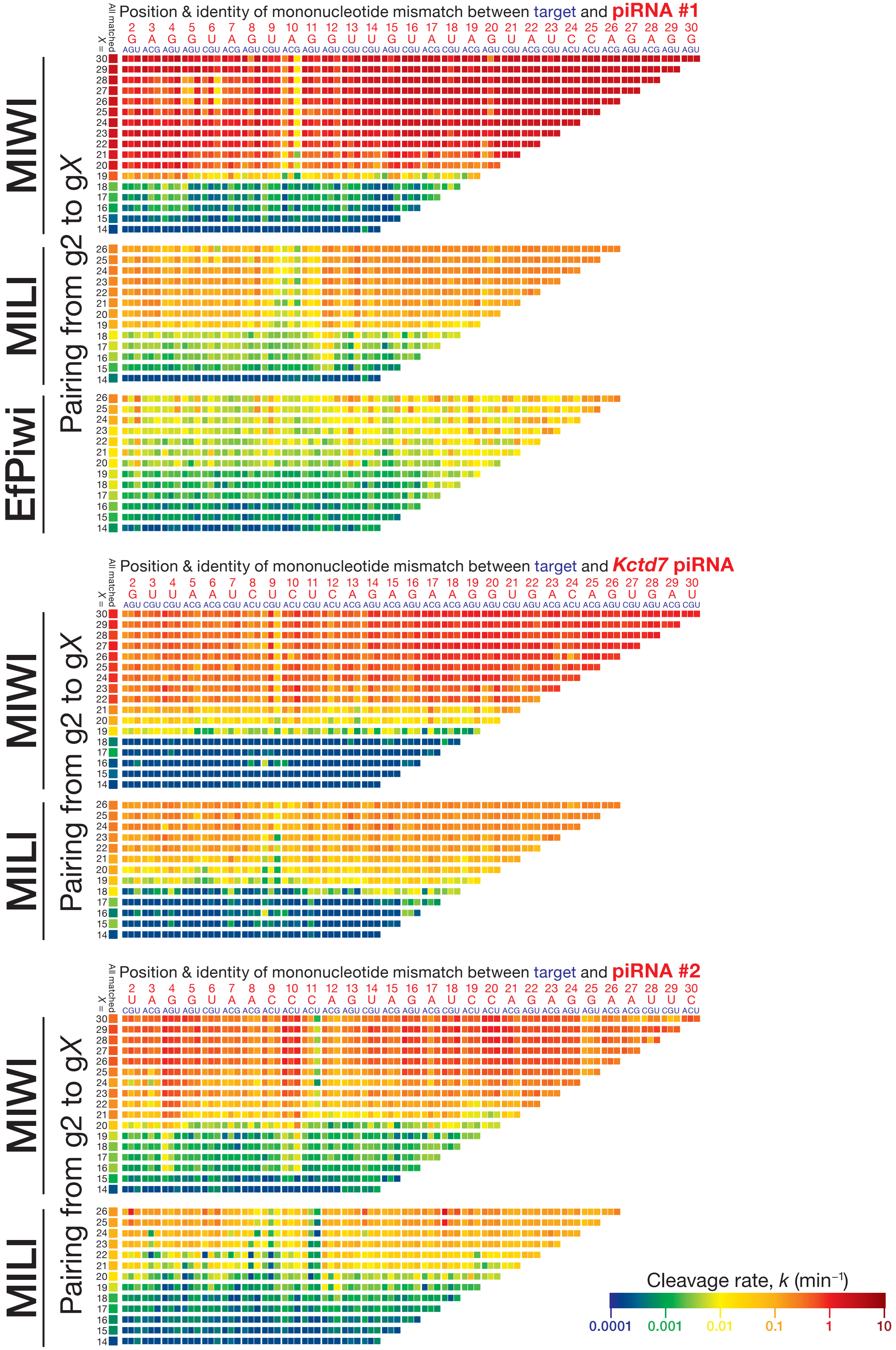


**Supplementary Figure 3.** MILI, MIWI, and EfPiwi pre-steady-state cleavage rates for piRNA #1, Kctd7 piRNA, or piRNA #2 targets with a single unpaired nucleotide.

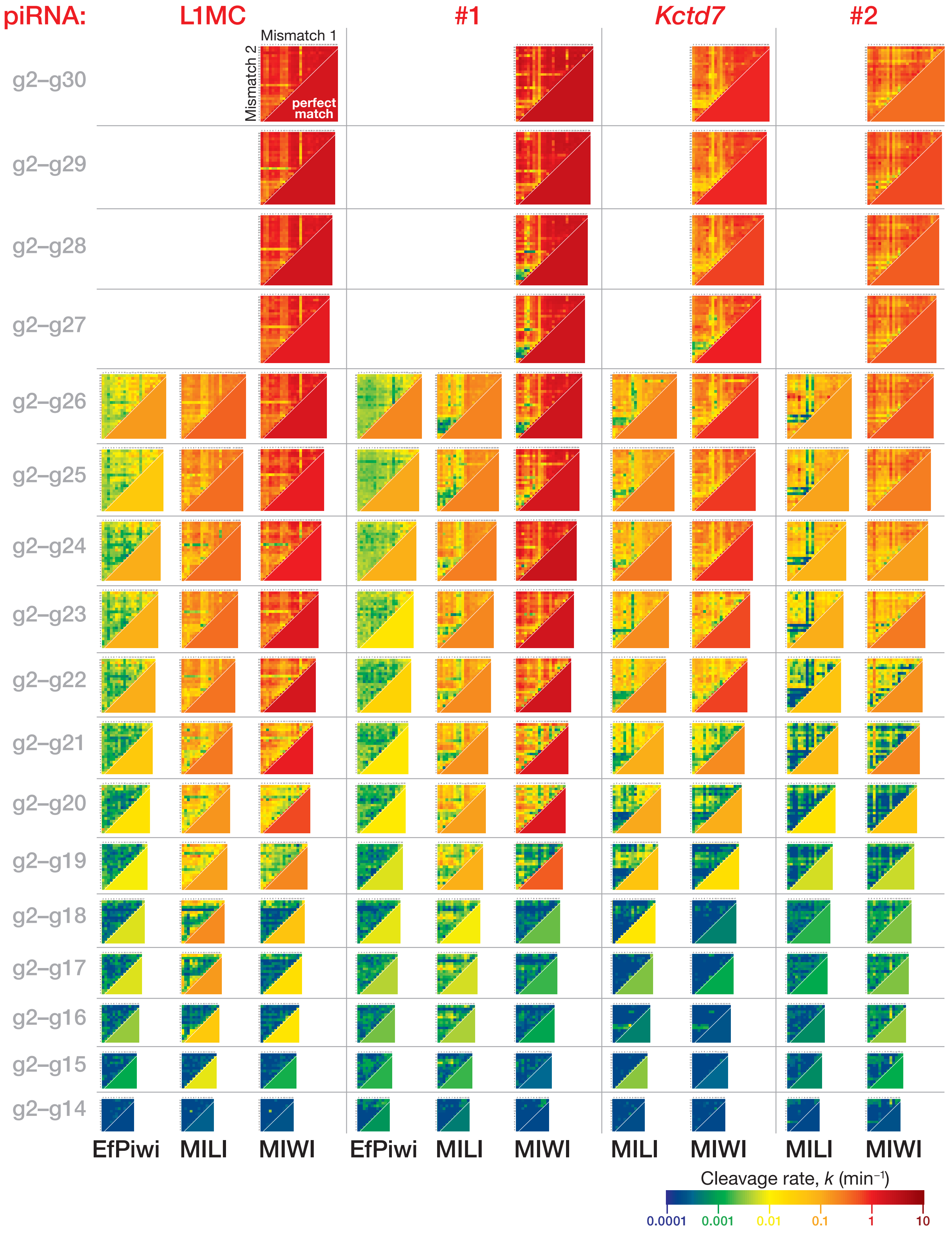


**Supplementary Figure 4.** MILI, MIWI, EfPiwi pre-steady-state cleavage rates for L1MC piRNA, piRNA #1, Kctd7 piRNA, or piRNA #2 targets with two unpaired nucleotides.

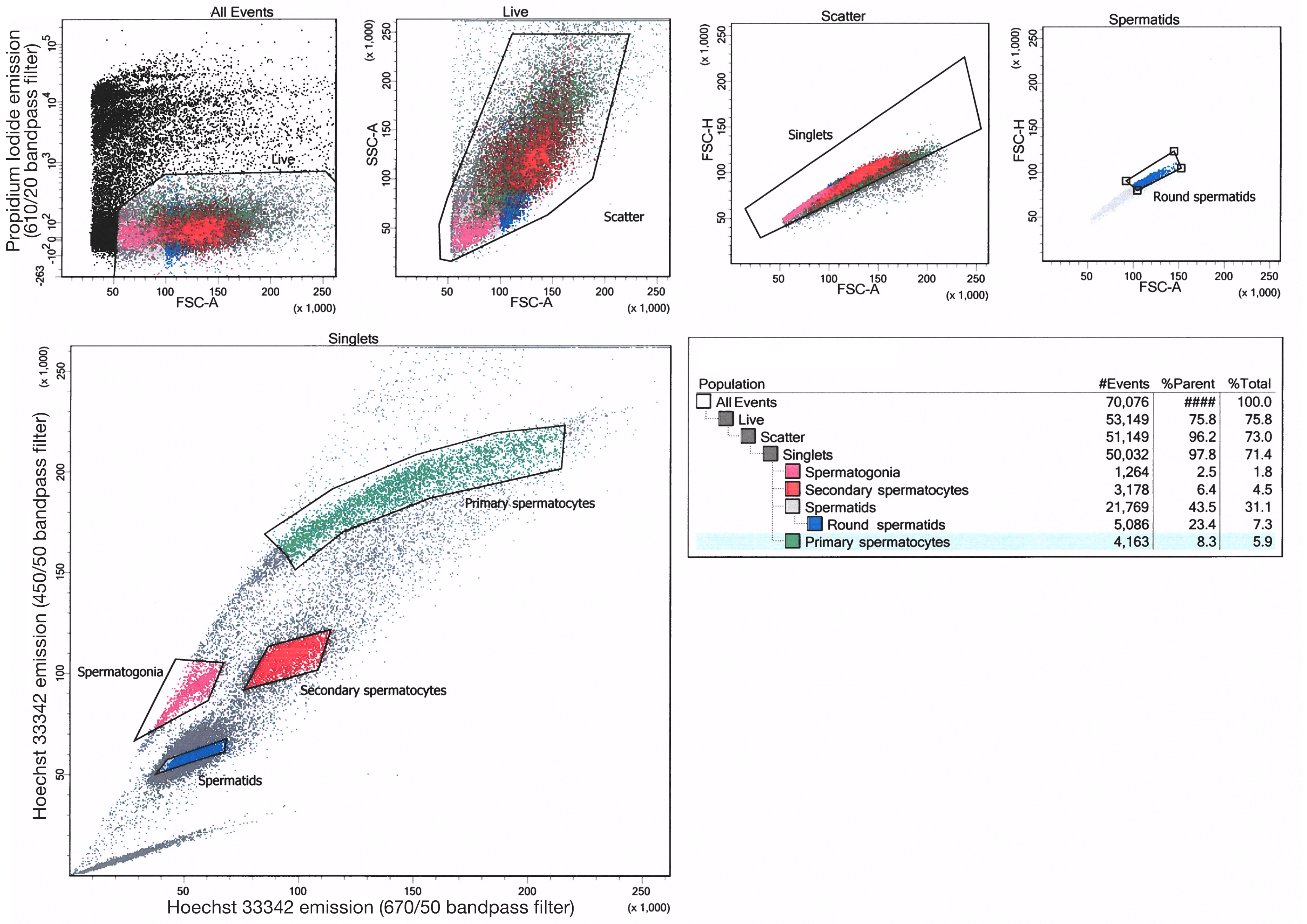


| Population | #Events | %Parent | %Total |
|---|---|---|---|
| All Events | 70,076 | #### | 100.0 |
| Live | 53,149 | 75.8 | 75.8 |
| Scatter | 51,149 | 96.2 | 73.0 |
| Singlets | 50,032 | 97.8 | 71.4 |
| Spermatogonia | 1,264 | 2.5 | 1.8 |
| Secondary spermatocytes | 3,178 | 6.4 | 4.5 |
| Spermatids | 21,769 | 43.5 | 31.1 |
| Round spermatids | 5,086 | 23.4 | 7.3 |
| Primary spermatocytes | 4,163 | 8.3 | 5.9 |

**Supplementary Figure 5.** FACS gating strategy to purify mouse primary germ cells
