# Supplementary material for: Relaxed targeting rules help PIWI proteins silence transposons
Source: Nature. 2023 Jun 21;619(7969):394–402. doi: 10.1038/s41586-023-06257-4 (PMC10338343; doi:10.1038/s41586-023-06257-4)
Supplement: Supplementary file 2 — Reporting Summary [file 41586_2023_6257_MOESM2_ESM.pdf]

nature portfolio

Corresponding author(s): Phillip D. Zamore

Last updated by author(s): May 5, 2023

Reporting Summary

Nature Portfolio wishes to improve the reproducibility of the work that we publish. This form provides structure for consistency and transparency in reporting. For further information on Nature Portfolio policies, see our Editorial Policies and the Editorial Policy Checklist.

Statistics

For all statistical analyses, confirm that the following items are present in the figure legend, table legend, main text, or Methods section.

n/a	Confirmed	
☐	☒	The exact sample size (*n*) for each experimental group/condition, given as a discrete number and unit of measurement
☐	☒	A statement on whether measurements were taken from distinct samples or whether the same sample was measured repeatedly
☐	☒	The statistical test(s) used AND whether they are one- or two-sided<br>*Only common tests should be described solely by name; describe more complex techniques in the Methods section.*
☒	☐	A description of all covariates tested
☐	☒	A description of any assumptions or corrections, such as tests of normality and adjustment for multiple comparisons
☐	☒	A full description of the statistical parameters including central tendency (e.g. means) or other basic estimates (e.g. regression coefficient) AND variation (e.g. standard deviation) or associated estimates of uncertainty (e.g. confidence intervals)
☐	☒	For null hypothesis testing, the test statistic (e.g. *F*, *t*, *r*) with confidence intervals, effect sizes, degrees of freedom and *P* value noted<br>*Give P values as exact values whenever suitable.*
☒	☐	For Bayesian analysis, information on the choice of priors and Markov chain Monte Carlo settings
☒	☐	For hierarchical and complex designs, identification of the appropriate level for tests and full reporting of outcomes
☐	☒	Estimates of effect sizes (e.g. Cohen's *d*, Pearson's *r*), indicating how they were calculated

Our web collection on statistics for biologists contains articles on many of the points above.

Software and code

Policy information about availability of computer code

Data collection	Typhoon FLA 7000; BD FACSAria II Cell Sorter; Illumina NextSeq 550; Illumina NovaSeq 6000.
Data analysis	python2 (v2.7.9) and python3 (v3.8.2) including the following modules: Trust Region Reflective algorithm in optimize.curve_fit from scipy (v1.8.1), RepeatedStratifiedKFold, cross_validate, and Limited-memory Broyden–Fletcher–Goldfarb–Shanno algorithm (L-BFGS) in LogisticRegression from scikit-learn; RNAplex from ViennaRNA (v2.5.1); fastx toolkit (v0.0.14); bowtie2 (v2.2.0); STAR (v2.3.1); StringTie (v1.3.4); DESeq2 (v1.18.1); bowtie (v1.0.0); SAMtools (v1.0.0); Microsoft Excel 2013; CRISPR design tool (crispr.mit.edu/); IgorPro 6.11 (WaveMetrics); Tailor (https://github.com/jhhung/Tailor); piPipes (https://github.com/bowhan/piPipes)

For manuscripts utilizing custom algorithms or software that are central to the research but not yet described in published literature, software must be made available to editors and reviewers. We strongly encourage code deposition in a community repository (e.g. GitHub). See the Nature Portfolio guidelines for submitting code & software for further information.

Data

Policy information about availability of data

All manuscripts must include a data availability statement. This statement should provide the following information, where applicable:

- Accession codes, unique identifiers, or web links for publicly available datasets
- A description of any restrictions on data availability
- For clinical datasets or third party data, please ensure that the statement adheres to our policy

Sequencing data are available from the National Center for Biotechnology Information Small Read Archive using accession number PRJNA848233. Code used in this work was deposited at github.com/ildargv/Gainetdinov_et_al_2023; mouse genome sequence and annotation (build mm10/GRCm38.92) were downloaded from https://ftp.ensembl.org/pub/release-92/fasta/mus_musculus/dna/ and https://ftp.ensembl.org/pub/release-92/gtf/mus_musculus/; transposon consensus sequences were obtained from Repbase (v27.02; https://www.girinst.org/repbase/).

Human research participants

Policy information about studies involving human research participants and Sex and Gender in Research.

Reporting on sex and gender	N/A
Population characteristics	N/A
Recruitment	N/A
Ethics oversight	N/A

Note that full information on the approval of the study protocol must also be provided in the manuscript.

Field-specific reporting

Please select the one below that is the best fit for your research. If you are not sure, read the appropriate sections before making your selection.

☒ Life sciences ☐ Behavioural & social sciences ☐ Ecological, evolutionary & environmental sciences

For a reference copy of the document with all sections, see nature.com/documents/nr-reporting-summary-flat.pdf

Life sciences study design

All studies must disclose on these points even when the disclosure is negative.

Sample size	No statistical method was used to determine the sample size. For biological samples, the maximum possible sample size (n = 4–12) was used for each type of data, ensuring that variability arising from all accountable sources was incorporated in the analyses (animal, day of data collection, reagent lots). For biochemical experiments, sample size was n = 3 to ensure reproducibility, i.e., for effect sizes of >2-fold, Relative Standard Deviation was <50% for >90% of data.
Data exclusions	No data were excluded from the analyses.
Replication	All data were collected during independent trials conducted on separate days. When using several types of data for analyses, all possible permutations of samples were analyzed (e.g., 4 control × 4 mutant data sets produced 16 permutations). All attempts at replication were successful.
Randomization	This study did not involve treatment or exposure of animals to any agent. Instead, the goal of this work was to compare untreated wild-type mice and untreated mutant mice lacking piRNAs from four genomic loci: all wild-type animals were compared to all mutant mice. Therefore, randomization is not relevant to this study.
Blinding	Blinding is not relevant to our study, because during analyses wild-type control and mutant data sets are easily identified. Blinding was not performed during data acquisition and/or analysis.

Reporting for specific materials, systems and methods

We require information from authors about some types of materials, experimental systems and methods used in many studies. Here, indicate whether each material, system or method listed is relevant to your study. If you are not sure if a list item applies to your research, read the appropriate section before selecting a response.

Materials & experimental systems

n/a	Involved in the study
☐	☒ Antibodies
☐	☒ Eukaryotic cell lines
☒	☐ Palaeontology and archaeology
☐	☒ Animals and other organisms
☒	☐ Clinical data
☒	☐ Dual use research of concern

Methods

n/a	Involved in the study
☒	☐ ChIP-seq
☐	☒ Flow cytometry
☒	☐ MRI-based neuroimaging

Antibodies

Antibodies used	Anti-FLAG antibody (M2, Sigma M8823); Anti-SCP3 antibody (Abcam, ab15093); Anti-phospho-Histone H2A.X (Ser139) antibody, clone JBW301 (Millipore, 05-636, clone JBW301); Donkey anti-Mouse IgG (H+L) Highly Cross-Adsorbed Secondary Antibody, Alexa Fluor 594 (ThermoFisher, A-21203); Donkey anti-Rabbit IgG (H+L) Highly Cross-Adsorbed Secondary Antibody, Alexa Fluor 488 (ThermoFisher, A-21206)
Validation	Anti-FLAG antibody (https://www.sigmaaldrich.com/content/dam/sigma-aldrich/docs/Sigma/Bulletin/f1804bul.pdf); Anti-SCP3 antibody (https://www.abcam.com/products/primary-antibodies/scp3-antibody-ab15093.pdf); Anti-phospho-Histone H2A.X (Ser139) antibody, clone JBW301 (https://www.emdmillipore.com/US/en/product/Anti-phospho-Histone-H2A.X-Ser139-Antibody-clone-JBW301,MM_NF-05-636#anchor_COA)

Eukaryotic cell lines

Policy information about cell lines and Sex and Gender in Research

Cell line source(s)	HEK293T and Sf9 cells (lab stock) were obtained from ATCC. Primary mouse spermatocytes were from male mice.
Authentication	The cell lines were not authenticated; the cell lines were only to produce recombinant proteins.
Mycoplasma contamination	Not tested.
Commonly misidentified lines (See ICLAC register)	No commonly misidentified cell lines were used in the study.

Animals and other research organisms

Policy information about studies involving animals; ARRIVE guidelines recommended for reporting animal research, and Sex and Gender in Research

Laboratory animals	C57BL/6 wild-type and mutant adult male mice.
Wild animals	The study did not involve wild animals.
Reporting on sex	Only males have testes.
Field-collected samples	No field-collected samples were used in the study.
Ethics oversight	(1) PI on IACUC protocol: Phillip D. Zamore<br>(2) Name of IACUC: UMass Medical School Institutional Animal Care and Use Committee<br>(3) IACUC Docket: A2222-17, "Investigation of mechanisms of small RNA function in vivo"

Note that full information on the approval of the study protocol must also be provided in the manuscript.

Flow Cytometry

Plots

Confirm that:

☒ The axis labels state the marker and fluorochrome used (e.g. CD4-FITC).

☒ The axis scales are clearly visible. Include numbers along axes only for bottom left plot of group (a 'group' is an analysis of identical markers).

☒ All plots are contour plots with outliers or pseudocolor plots.

☒ A numerical value for number of cells or percentage (with statistics) is provided.

Methodology

Sample preparation	Testes of 2–6-month-old mice were isolated, decapsulated, and incubated for 15 min at 33°C in 1× Gey's Balanced Salt Solution (GBSS, Sigma, G9779) containing 0.4 mg/ml collagenase type 4 (Worthington, LS004188) rotating at 150 rpm. Seminiferous tubules were then washed twice with 1× GBSS and incubated for 15 min at 33°C in 1× GBSS with 0.5 mg/ml Trypsin and 1 μg/ml DNase I, rotating at 150 rpm. Next, tubules were homogenized by pipetting through a glass Pasteur pipette for 3 min at 4°C. Fetal bovine serum (FBS; 7.5% f.c., v/v) was added to inactivate trypsin, and the cell suspension was then strained through a pre-wetted 70 μm cell strainer (ThermoFisher, 22363548); cells were collected by centrifugation at 300 × g for 10 min. The supernatant was removed, cells resuspended in 1× GBSS containing 5% (v/v) FBS, 1 μg/ml DNase I, and 5 μg/ml Hoechst 33342 (ThermoFisher, 62249) and rotated at 150 rpm for 45 min at 33ºC. Propidium iodide (0.2 μg/ml, f.c.; ThermoFisher, P3566) was added, and cells strained through a pre-wetted 40 μm cell strainer (ThermoFisher, 22363547).
Instrument	FACSAria II Cell Sorter (BD Biosciences; UMass Medical School FACS Core)
Software	BD FACSDiva (v9.0)
Cell population abundance	Spermatogonia: ~100,000 cells/animal; ~95–100% pure with ≤ 5% pre-leptotene spermatocytes;<br>Primary spermatocytes: ~1,000,000 cells/animal; ~10–15% leptotene/zygotene spermatocytes, ~45–50% pachytene spermatocytes, ~35–40% diplotene spermatocytes;<br>Secondary spermatocytes: ~1,000,000 cells/animal; ~100%;<br>Round spermatids: ~1,500,000 cells/animal; ~95–100%, ≤ 5% elongated spermatids.
Gating strategy	The gating strategy used to sort mouse primary germ cells is detailed in Supplementary Figure 5. Briefly, propidium iodide was used to label dead cells (top left panel in Supplementary Figure 5), forward and side scatter were used to isolate single cells (two top middle panels in Supplementary Figure 5), Hoechst 33342 emission in 450/50 and 670/50 bandpass filters was used to separate spermatogonia, spermatocytes, and spermatids (bottom left panel in Supplementary Figure 5). Forward scatter was then used to isolate round spermatids form the mixed population of round and elongated spermatids top right panel in Supplementary Figure 5). The percentages for each subpopulation are shown in the bottom right panel in Supplementary Figure 5.

☒ Tick this box to confirm that a figure exemplifying the gating strategy is provided in the Supplementary Information.

March 2021
