# Supplementary material for: Relaxed targeting rules help PIWI proteins silence transposons
Source: Nature. 2023 Jun 21;619(7969):394–402. doi: 10.1038/s41586-023-06257-4 (PMC10338343; doi:10.1038/s41586-023-06257-4)
Supplement: Supplementary file 5 — Supplementary Tables 2–4, 6 and 7. Source data for analyses of in vivo cleavage by MILI and MIWI (number of putative cleavage sites). Mouse strains and sequences of oligonucleotides used in this study. Amount of spike-in RNAs used to prepare small RNA-seq and RNA-seq libraries in this study. [file 41586_2023_6257_MOESM5_ESM.pdf]

Supplementary Table 2. Total number of “*pi2*, *pi9*, *pi17* piRNA:cleavage site” pairs for which the abundance of cleavage product decreased >8-fold in *pi2*$^{-/-}$; *pi9*$^{-/-}$; *pi17*$^{-/-}$ triple mutant mice compared to wild-type controls.

**Contiguous pairing** (Fig. 3b)	
g2g10	444,728
g2g11	192,056
g2g12	61,309
g2g13	19,869
g2g14	9,445
g2g15	2,756
g2g16	2,014
g2g17	862
g2g18	257
g2g19	225
g2g20	152
**Mononucleotide mismatches** (Fig. 3c and Extended Data Fig. 8a)	
g2g16, mismatch at g2	2,153
g2g16, mismatch at g3	1,173
g2g16, mismatch at g4	848
g2g16, mismatch at g5	3,020
g2g16, mismatch at g6	842
g2g16, mismatch at g7	1,111
g2g16, mismatch at g8	1,146
g2g16, mismatch at g9	1,123
g2g16, mismatch at g10	1,091
g2g16, mismatch at g11	1,160
g2g16, mismatch at g12	1,456
g2g16, mismatch at g13	1,262
g2g16, mismatch at g14	1,197
g2g16, mismatch at g15	1,280
g2g16, mismatch at g16	5,388
g2g17, mismatch at g2	592
g2g17, mismatch at g3	311
g2g17, mismatch at g4	322
g2g17, mismatch at g5	323
g2g17, mismatch at g6	263
g2g17, mismatch at g7	225

g2g17, mismatch at g8	304
g2g17, mismatch at g9	376
g2g17, mismatch at g10	364
g2g17, mismatch at g11	436
g2g17, mismatch at g12	445
g2g17, mismatch at g13	539
g2g17, mismatch at g14	423
g2g17, mismatch at g15	543
g2g17, mismatch at g16	612
g2g17, mismatch at g17	1,526
g2g18, mismatch at g2	235
g2g18, mismatch at g3	121
g2g18, mismatch at g4	173
g2g18, mismatch at g5	179
g2g18, mismatch at g6	84
g2g18, mismatch at g7	158
g2g18, mismatch at g8	103
g2g18, mismatch at g9	160
g2g18, mismatch at g10	174
g2g18, mismatch at g11	193
g2g18, mismatch at g12	258
g2g18, mismatch at g13	197
g2g18, mismatch at g14	175
g2g18, mismatch at g15	270
g2g18, mismatch at g16	210
g2g18, mismatch at g17	340
g2g18, mismatch at g18	600
g2g19, mismatch at g2	143
g2g19, mismatch at g3	58
g2g19, mismatch at g4	53
g2g19, mismatch at g5	30
g2g19, mismatch at g6	48
g2g19, mismatch at g7	23
g2g19, mismatch at g8	77
g2g19, mismatch at g9	61
g2g19, mismatch at g10	85
g2g19, mismatch at g11	120

g2g19, mismatch at g12	144
g2g19, mismatch at g13	62
g2g19, mismatch at g14	80
g2g19, mismatch at g15	66
g2g19, mismatch at g16	164
g2g19, mismatch at g17	56
g2g19, mismatch at g18	254
g2g19, mismatch at g19	210
**Mononucleotide target indels** (Extended Data Fig. 9a)	
g2g17, target deletion between g3 and g4	113
g2g17, target deletion between g4 and g5	97
g2g17, target deletion between g5 and g6	124
g2g17, target deletion between g6 and g7	367
g2g17, target deletion between g7 and g8	106
g2g17, target deletion between g8 and g9	97
g2g17, target deletion between g9 and g10	97
g2g17, target deletion between g10 and g11	124
g2g17, target deletion between g11 and g12	211
g2g17, target deletion between g12 and g13	136
g2g17, target deletion between g13 and g14	114
g2g17, target deletion between g14 and g15	195
g2g17, target deletion between g15 and g16	190
g2g17, target deletion between g16 and g17	190
g2g17, target insertion between g2 and g3	353
g2g17, target insertion between g3 and g4	173
g2g17, target insertion between g4 and g5	117
g2g17, target insertion between g5 and g6	173
g2g17, target insertion between g6 and g7	138
g2g17, target insertion between g7 and g8	119
g2g17, target insertion between g8 and g9	99
g2g17, target insertion between g9 and g10	120
g2g17, target insertion between g10 and g11	97
g2g17, target insertion between g11 and g12	192
g2g17, target insertion between g12 and g13	304
g2g17, target insertion between g13 and g14	168
g2g17, target insertion between g14 and g15	201
g2g17, target insertion between g15 and g16	306

g2g17, target insertion between g16 and g17	227
**Contiguous pairing** (Fig. 3e)	
g2g15, piRNA <30 pM	1,192
g2g15, piRNA 30–50 pM	365
g2g15, piRNA 50–100 pM	467
g2g15, piRNA 100–500 pM	598
g2g15, piRNA >500 pM	134
g3g16, piRNA <30 pM	498
g3g16, piRNA 30–50 pM	347
g3g16, piRNA 50–100 pM	310
g3g16, piRNA 100–500 pM	927
g3g16, piRNA >500 pM	67
g4g17, piRNA <30 pM	464
g4g17, piRNA 30–50 pM	186
g4g17, piRNA 50–100 pM	177
g4g17, piRNA 100–500 pM	264
g4g17, piRNA >500 pM	20
g5g18, piRNA <30 pM	394
g5g18, piRNA 30–50 pM	257
g5g18, piRNA 50–100 pM	233
g5g18, piRNA 100–500 pM	230
g5g18, piRNA >500 pM	66

Supplementary Table 3. Mouse strains used in this study.

Strain	Two guide RNAs sequences	Deletion coordinates (mm10)	Genotyping mutant allele		Genotyping wild-type allele	
			Primers	Amplicon size	Primers	Amplicon size
pi2$^{-/-}$ (*pi2*$^{em1Pdz/\ em1Pdz}$)	GCT TGA TCG TCA GGG ACT AA<br><br>TCA GAG GCT AAG TCC CAT TA	chr2:92539403–92542146	CCA CCT CCA GCT CTT CCT CT<br><br>TTA GCT GCC TCA AGA GTG GC	Mut 732 bp	CCC TTG ATC ATA CCC ACC TCC<br><br>TGT CAA CAA ACC CCC AGG AC	501 bp
pi7$^{-/-}$ (*pi7*$^{em1Pdz/\ em1Pdz}$)	CCG GGG CCT GCA AAG AAG AA<br><br>GAC CAC CCT GAA ACC TGT AA	chr7:73816369-73816663	CAT GT CGT TGC TGG GCA AAA<br><br>GTG GAC CTG TTG CAG GAA CT	WT 983 bp<br>Mut 664 bp	CCC TTT GCC TAG GAC TGT GG<br><br>CAT GTC GTT GCTG GGC AAA A	488 bp
pi9$^{-/-}$ (*pi9*$^{em1Pdz/\ em1Pdz}$)	GGC CTG CAG CAT GCT CTT GC<br><br>GTT TAG GGT TTG GGT AAG TT	chr9:67733702–67734069	AGA TCC AGA GGC AGG CTT TT<br><br>TGC CAG CT CTC TTG TCA GAA	WT 774 bp<br>Mut 393 bp	CGT GGA CAA CAG GGA CAC TA<br><br>CCA CCC CAA ATG CCA TGA AG	307 bp
pi17$^{-/-}$ (*pi17*$^{em1Pdz/\ em1Pdz}$) reported in Ref.[10]; MGI 6441981	GTC CCT TCA CAC GGC CGT TTA<br><br>GCT CTG TCT GAC AAC GGG AC	chr17:27324887–27325439	CGC AGC CCA TCC ATT TCT TG<br><br>GAC TAG CGC CAG TTT CCA CT	WT 1000 bp<br>Mut 44 8bp	AGG TCT GCA CGT AGT CTC CT<br><br>GGG TGT GGC CAC ATG TAT CA	368 bp

Supplementary Table 4. Sequences of oligonucleotides used in this study

	Name	Sequence (5′-to-3′)	Notes
piRNA synthetic guides	piRNA #1 in MIWI	/phos/UGAGGUAGUAGGUUGUAUAGUAUCCAGAGG	5′ monophosphorylated RNA
	piRNA #1 in MILI	/phos/UGAGGUAGUAGGUUGUAUAGUAUCCA	
	piRNA #1 in AGO2	/phos/UGAGGUAGUAGGUUGUAUAGU	
	L1MC piRNA in MIWI	/phos/UAACUAAAUACUAUGCAAGCUGUAGGUCCU	
	L1MC piRNA in MILI	/phos/UAACUAAAUACUAUGCAAGCUGUAGG	
	Kctd7 piRNA in MIWI	/phos/UGUUAAUCUCUCAGAGAAGGUGACAGUGAU	
	Kctd7 piRNA in MILI	/phos/UGUUAAUCUCUCAGAGAAGGUGACAG	
	piRNA # 2 in MIWI	/phos/UUAGGUAACCCAGUAGAUCCAGAGGAAUUC	
	piRNA # 2 in MILI	/phos/UUAGGUAACCCAGUAGAUCCAGAGGA	
Capture oligonucleotides	piRNA # 1	Bio-mAmUmA mGmAmC mUmGmC mGmAmC mAmAmU mAmGmC mCmUmA mCmCmU mCmCmG mAmAmC mGmGmC mGmAmG	5′ biotinylated fully 2′-O-methylated RNA complementary to piRNA nucleotides g2–g8 and g13–g16
	L1MC piRNA	Bio-mAmAmA mAmAmA mAmAmA mAmUmG mAmGmA mGmGmC mAmUmA mCmAmC mUmUmU mAmGmU mUmAmC mUmGmC	
	Kctd7 piRNA	Bio-mAmAmG mGmAmA mCmAmG mAmAmG mAmGmA mGmCmU mCmUmA mCmAmC mGmAmU mUmAmA mCmAmC mUmGmC	
	piRNA #2	Bio-mAmCmU mAmCmU mGmCmA mGmCmA mCmAmA mCmCmC mUmAmC mCmAmA mAmUmU mAmCmC mUmAmA mCmUmG mC	
Competitor oligonucleotides	piRNA # 1	Bio-CTCGCCGTTCGGAGGTAGGCTATTGTCGCAGTCTAT	5′ biotinylated DNA fully complementary to capture oligonucleotide
	L1MC piRNA	Bio-GCAGTAACTAAAGTGTATGCCTCTCATTTTTTTTTT	
	Kctd7 piRNA	Bio-GCAGTGTTAATCGTGTAGAGCTCTCTTCTGTTCCTT	
	piRNA #2	Bio-GCAGTTAGGTAATTTGGTAGGGTTGTGCTGCAGTAGT	
Primers for PCR amplification of targets for cleavage assays	Forward primer	GCGTAATACGACTCACTATAGGGTTTTAATGAATACGATTT	DNA primers used with pGL2 plasmid as the template to amplify dsDNA for T7 in vitro transcription of cleavage targets; g2–g30 fully complementary target site is underlined
	Reverse primer for piRNA #1 target	ACACTATAGATTTATACCTAGTTAAACAGCGGAACTGTGTA TAAAAGGTTGAGGTAGTAGGTTGTATAGTATCCAGAGGATA GGTCTCCAATTCATTATCAGTGCAAT	
	Reverse primer for L1MC piRNA target	ACACTATAGATTTATACCTAGTTAAACAGCGGAACTGTGTA TAAAAGGTTAACTAAATACTATGCAAGCTGTAGGTCCTATA GGTCTCCAATTCATTATCAGTGCAAT	
	Reverse primer for Kctd7 piRNA target	ACACTATAGATTTATACCTAGTTAAACAGCGGAACTGTGTA TAAAAGGTTGTTAATCTCTCAGAGAAGGTGACAGTGATATA GGTCTCCAATTCATTATCAGTGCAAT	

	Reverse primer for piRNA #2 target	ACACTATAGATTTATACCTAGTTAAACAGCGGAACTGTGTA<br>TAAAAGGTTTAGGTAACCCAGTAGATCCAGAGGAATTCATA<br>GGTCTCCAATTCATTATCAGTGCAAT	
RBNS primers and oligos	Target library	GAGUUCUACAGUCCGACGAUCNNNNNNNNNNNNNNNNNNN<br>UGGAAUUCUCGGGUGCCAA	RNA
	RT primer	CCTTGGCACCCGAGAATTCCA	DNA
	Forward (P5) primer	AATGATACGGCGACCACCGAGATCTACACGTTC AGAGTTCTACAGTCCGA	DNA primers for amplification of RBNS library, XXXXXX represents 6-nt sequencing barcode
	Reverse (P7) primer	CAAGCAGAAGACGGCATACGAGATXXXXXX<br>GTGACTGGAGTTCCTTGGCACCCGAGAATTCCA	
	DNA blocking oligos	TTGGCACCCGAGAAT	DNA
		GTCGGACTGTAGAACTC	
Targets used in filter binding assay for piRNA #1 in MIWI	piRNA #1 g2–g10	/phos/AAAAAAAAAAAAAAAAAAAUACUACCUCA	5′ monophosphorylated RNA
	piRNA #1 g8–g16	/phos/AAAAAAAAAAAAAAACAACCUACAAAAAAA	
CNS primers and oligos	Forward primer	GCGTAATACGACTCACTATAGG GTTCAGAGTTCTACAGTCCGACGATC	DNA primers for adding T7 promoter to TWIST Bioscience DNA oligo pool of CNS targets
	Reverse primer	CCTTGGCACCCGAGAATTCCA	
	DNA blocking oligos	CCTTGGCACCCGAGAA	DNA
		TCGGACTGTAGAACTCTGAAC	
	RT primer	CCTTGGCACCCGAGAATTCCA	DNA
	Forward (P5) primer	AATGATACGGCGACCACCGAGATCTACACGTTC AGAGTTCTACAGTCCGA	DNA primers for final amplification of CNS library, XXXXXX represents 6-nt sequencing barcode
	Reverse (P7) primer	CAAGCAGAAGACGGCATACGAGATXXXXXX<br>GTGACTGGAGTTCCTTGGCACCCGAGAATTCCA	
	Equimolar mix of five spike-in RNA oligonucleotides	/phos/UCGUGGAUGUCGUACGUAUCUGGAAUUCUCGGGUGCCAAGG<br>/phos/UGCUGGAUGUCCAACGUAUCUGGAAUUCUCGGGUGCCAAGG<br>/phos/ACCUGGAUGUGGAACGUAUCUGGAAUUCUCGGGUGCCAAGG<br>/phos/CGAACGUAUCUAUUUACAAAUGGAAUUCUCGGGUGCCAAGG<br>/phos/CGAACGUAUCAUAUUAGUUAUGGAAUUCUCGGGUGCCAAGG	5′ monophosphorylated RNA
Small RNA sequencing adapters, oligos, and primers	Equimolar mix of nine spike-in RNA oligonucleotides	/phos/UGCUAGUCUGUUAUCGACCUGACCUCAUAG	5′ monophosphorylated RNA
		/phos/UGCUAGUCUGUUCGAUACCUGACCUCAUAG	
		/phos/UGCUAGUCUGUUGUCACGAAGACCUCAUAG	
		/phos/UGCUAGUCUUAUCGACCUCCUCAUAG	
		/phos/UGCUAGUCUUCGAUACCUCCUCAUAG	
		/phos/UGCUAGUCUUGUCACGAACCUCAUAG	
		/phos/UGCUAGUUAUCGACCUUCAUAG	
		/phos/UGCUAGUUCGAUACCUUCAUAG	

		/phos/UGCUAGUUGUCACGAAUCAUAG	
	3′ DNA adapter	/rApp/NNNGTCNNNTAGNNNTGGAATTCTCGGGTGCCAAGG/ddC/	5′ adenylated, 3′ dideoxycytosine blocked DNA adapter
	Equimolar mix of two 5′ RNA adaptors	GUUCAGAGUUCUACAGUCCGACGAUCNNNCGANNNUACNNN<br>GUUCAGAGUUCUACAGUCCGACGAUCNNNAUCNNNAGUNNN	RNA
	RT primer	CCTTGGCACCCGAGAATTCCA	DNA
	Forward (P5) primer	AATGATACGGCGACCACCGAGATCTACACGTTC AGAGTTCTACAGTCCGA	DNA primers for final amplification of CNS library, XXXXXX represents 6-nt sequencing barcode
	Reverse (P7) primer	CAAGCAGAAGACGGCATACGAGATXXXXXX GTGACTGGAGTTCCTTGGCACCCGAGAATTCCA	
Three sets of unique molecular identifier (UMI) containing RNA-seq adapters	Adapter set 1	/phos/CCCNNNNNAGATCGGAAGAGCACACGTCT<br>ACACTCTTTCCCTACACGACGCTCTTCCGATCTNNNNNGGGT	Two adapters in each set are first annealed to each other in three separate tubes, then the three annealed sets are equimolarly mixed to a final total concentration of 3.3 µM each duplex
	Adapter set 2	/phos/GATNNNNNAGATCGGAAGAGCACACGTCT<br>ACACTCTTTCCCTACACGACGCTCTTCCGATCTNNNNNATCT	
	Adapter set 3	/phos/TGANNNNNAGATCGGAAGAGCACACGTCT<br>ACACTCTTTCCCTACACGACGCTCTTCCGATCTNNNNNTCAT	
Adapters and primers for cloning and sequencing long 5′ mono-phosphorylated RNAs	Equimolar mix of two 5′ RNA adaptors	GUUCAGAGUUCUACAGUCCGACGAUCNNNCGANNNUACNNN<br>GUUCAGAGUUCUACAGUCCGACGAUCNNNAUCNNNAGUNNN	RNA
	RT primer	GCACCCGAGAATTCCANNNNNNNN	DNA
	PCR 1 forward primer	CTACACGTTCAGAGTTCTACAGTCCGA	DNA primers for the first PCR amplification
	PCR 1 reverse primer	GCCTTGGCACCCGAGAATTCCA	
	Forward (P5) primer	AATGATACGGCGACCACCGAGATCTACACGTTC AGAGTTCTACAGTCCGA	DNA primers for the second PCR amplification
	Reverse (P7) primer	CAAGCAGAAGACGGCATACGAGATXXXXXX GTGACTGGAGTTCCTTGGCACCCGAGAATTCCA	

Supplementary Table 6. Number of Primary Spermatocytes and Amount of Spike-In Mix Used to Prepare Small RNA Sequencing Libraries

Genotype	Trial	Cell number	Amount of spike-in, attomol
C57BL/6	Rep1	31,400	370
	Rep2	68,900	4000
	Rep3	47,200	3000
	Rep4	89,100	4000
	Rep5	48,900	3000
	Rep6	60,300	4000
	Rep7	63,100	4000
	Rep8	99,700	4000
	Rep9	112,500	4000
	Rep10	116,400	4000
	Rep11	137,000	4000
	Rep12	112,000	4000
pi2$^{-/-}$; *pi9*$^{-/-}$; *pi17*$^{-/-}$	Rep1	47,800	4000
	Rep2	43,700	4000
	Rep3	195,000	4000
	Rep4	167,300	4000
	Rep5	93,800	4000
	Rep6	143,000	4000
	Rep7	129,900	4000
	Rep8	77,400	4000
	Rep9	80,600	4000
pi2$^{-/-}$	Rep1	44,400	4000
pi7$^{-/-}$	Rep1	63,600	4000
	Rep2	122,800	4000
	Rep3	76,000	4000
pi9$^{-/-}$	Rep1	42,700	4000
pi17$^{-/-}$	Rep1	91,300	4000

Supplementary Table 7. Number of Primary Spermatocytes and Amount of ERCC Mix Used to Prepare RNA Sequencing Libraries

**Genotype**	**Replicate**	**Cell number**	**Amount of spike-in, attomole (1 µl of 1/100 dilution of ERCC Mix 1)**
C57BL/6	Rep1	103,800	1035.15
	Rep2	62,100	1035.15
	Rep3	67,400	1035.15
	Rep4	59,100	1035.15
	Rep5	102,600	1035.15
	Rep6	96,300	1035.15
pi2$^{-/-}$; *pi9*$^{-/-}$; *pi17*$^{-/-}$	Rep1	98,400	1035.15
pi2$^{-/-}$	Rep1	89,200	1035.15
pi7$^{-/-}$	Rep1	191,700	1035.15
pi9$^{-/-}$	Rep1	63,100	1035.15
pi17$^{-/-}$	Rep1	111,900	1035.15
